# Natural infection of murine norovirus in conventional and specific pathogen-free laboratory mice

**DOI:** 10.3389/fmicb.2013.00012

**Published:** 2013-01-30

**Authors:** Takeo Ohsugi, Kumi Matsuura, Satomi Kawabe, Naoko Nakamura, Jerald M. Kumar, Makoto Wakamiya, Saki Morikawa, Toru Urano

**Affiliations:** ^1^Division of Microbiology and Genetics, Institute of Resource Development and Analysis, Kumamoto UniversityKumamoto, Japan; ^2^Centre for Cellular and Molecular BiologyHyderabad, India

**Keywords:** genetically modified mice, mice, microbiological monitoring, MNV, mouse norovirus, specific pathogen-free

## Abstract

Noroviruses cause most cases of acute viral gastroenteritis worldwide. The lack of a cell culture infection model for human norovirus necessitates the use of molecular methods and/or viral surrogate models amenable to cell culture to predict norovirus inactivation. Murine norovirus (MNV) may be used to construct a small animal model for studying the biology and pathogenesis of noroviruses because MNV is the only norovirus that replicates in cell culture and a small animal model. However, recent studies have shown that natural MNV infection is widespread in laboratory mouse colonies. We investigated MNV infection in both conventional and specific pathogen-free (SPF) genetically modified mice from Japan and the US, and commercial mice from several animal breeders in Japan, using serological and molecular techniques. MNV antibodies were detected in 67.3% of conventional mice and 39.1% of SPF mice from Japan and 62.5% of conventional mice from the US. MNV antibodies were also found in 20% of commercial SPF C57BL/6 mice from one of three breeders. Partial gene amplification of fecal isolates from infected animals showed that the isolates were homologous to reported MNV sequences. These results suggest that both conventional and SPF laboratory mice, including commercial mice, are widely infected with MNV, which might require considerable attention as an animal model of human disease.

## INTRODUCTION

Human noroviruses are the major cause of non-bacterial epidemic gastroenteritis worldwide (Patel et al., 2009). The study of human norovirus has been hampered by the lack of a cell culture system. Murine norovirus (MNV) was first isolated and characterized as a sporadic and lethal pathogen in immunocompromised knockout mice (Karst et al., 2003). MNV is the only norovirus that replicates in cell culture and in a small animal model, though several studies have reported norovirus infection in humans, cattle, swine, dogs, and mice (Wobus et al., 2006; Patel et al., 2009). Thus, MNV is expected to be a surrogate for evaluating the resistance of human norovirus to disinfectants and can be used in animal model studies of human norovirus infection.

Natural MNV infection is prevalent in animal facilities around the world (Hsu et al., 2005; Perdue et al., 2007; Pritchett-Corning et al., 2009). These infections might influence not only the results of a mouse model for studying the biology and pathogenesis of noroviruses, but also those of other biological studies (Lencioni et al., 2008; Cadwell et al., 2010). Until recently, no MNV infection had been reported in laboratory mice in Japan. However, the first reports of MNV detected in conventional mouse colonies in Japan were published in 2009 (Goto et al., 2009b; Kitajima et al., 2009). Recent serological analysis of MNV found that MNV infection is also prevalent in conventional animal colonies in Japan (Kitagawa et al., 2010). However, information on the prevalence of MNV infection in specific pathogen-free (SPF) mice colonies and commercial SPF mice in Japan is not currently available. Most researchers in Japan think that MNV infection is absent among SPF mice facilities.

The Institute of Resource Development and Analysis at Kumamoto University collects, preserves, and distributes experimental animals; thus, numerous mice are transferred to the institute from across Japan and from the other countries for embryo freezing or microbiological cleaning (Nakagata and Yamamura, 2009). Our division in the institute checks the microbiological status of genetically modified mice before or after embryo transfer. In the present study, we examined the prevalence of MNV infection in conventional and SPF genetically modified mice and commercial SPF mice using both serological and molecular biological methods.

## MATERIALS AND METHODS

### MICE

Ninety-six genetically modified mice derived from 29 facilities in 2011, including 18 mice derived from five different animal facilities in the US which were to be converted to SPF grade by *in vitro* fertilization, were used in this study. Thirty commercial SPF C57BL/6 mice derived from three breeders in Japan were tested for MNV infection. After arriving at our facility, the mice were kept in the laminar flow racks (negative air pressure) in our quarantine animal rooms, which are separate from the SPF animal rooms, until the collection of embryos and sperm. The quarantine period was less than 1 week, except for five mice used in an experiment for the frequency of detection of MNV. All procedures involving animals and their care were approved by the Animal Care Committee of Kumamoto University in accordance with the Regulations for Animal Experiments at Kumamoto University.

### MICROBIOLOGICAL MONITORING

The animals were checked for the presence of specific pathogens using routine methods described previously (Goto et al., 2009a). Briefly, the analysis included a range of viruses, bacteria, mycoplasmas, and protozoans, which are listed in full in **Table 1**. The absence of all tested pathogens indicated that an animal was SPF grade.

**Table 1:** Microbiological status in tested facilities.

Pathogens	Methods*	U.S. facilities		Domestic facilities		Breeders		
		SPF^†^	Conv^†^	SPF	Conv	A	B	C
**Viruses**								
Mouse hepatitis virus	E, I	0/2^‡^	0/16	0/23	0/55	0/10	0/10	0/10
Sendai virus	E, I	0/2	0/16	0/23	0/55	0/10	0/10	0/10
**Bacteria and mycoplasma**								
*Citrobacter rodentium*	C	0/2	0/16	0/23	0/55	0/10	0/10	0/10
*Clostridium piliforme*	E, I	0/2	0/16	0/23	0/55	0/10	0/10	0/10
*Corynebacterium*	C	0/2	0/16	0/23	0/55	0/10	0/10	0/10
*Helicobacter hepaticus*	RT-PCR	0/2	0/16	0/23	0/55	0/10	0/10	0/10
*Mycoplasma pulmonis*	C, E, I	0/2	0/16	0/23	**1/55**	0/10	0/10	0/10
*Pasteurella*	C	0/2	0/16	0/23	0/55	0/10	0/10	0/10
*Salmonella* spp.	C	0/2	0/16	0/23	0/55	0/10	0/10	0/10
**Parasites and protozoa**								
*Aspiculuris tetraptera*	M	0/2	0/16	0/23	**23/55**	0/10	0/10	0/10
*Syphacia* spp.	M	0/2	0/16	0/23	**6/55**	0/10	0/10	0/10
*Giardia muris*	M	0/2	0/16	0/23	0/55	0/10	0/10	0/10
*Spironucleus muris*	M	0/2	0/16	0/23	0/55	0/10	0/10	0/10
*Trichomonas *spp.	M	0/2	**16/16**	0/23	**12/55**	0/10	0/10	0/10
*Octomitus pulcher*	M	0/2	0/16	0/23	**1/55**	0/10	0/10	0/10
*Entamoeba* spp.	M	0/2	0/16	0/23	**6/55**	0/10	0/10	0/10
*Chilomastix* spp.	M	0/2	0/16	0/23	**3/55**	0/10	0/10	0/10
Ectoparasite	M	0/2	0/16	0/23	**5/55**	0/10	0/10	0/10

* C, culture; E, ELISA; I, indirect fluorescent antibody method; M, microscopic examination; RT-PCR, reverse transcriptase-polymerase chain reaction.

† SPF, specific pathogen-free; Conv, conventional.

‡ Number of positive/number of tested. Positive results are indicated in bold.

### SEROLOGICAL TEST FOR MNV

Serological analysis of MNV infection was performed using an enzyme-linked immunosorbent assay (ELISA) kit (Mouse Norovirus; Biotech Trading Partners, Encinitas, CA, USA) according to the manufacturer’s protocol.

### RNA ISOLATION AND REVERSE TRANSCRIPTASE-POLYMERASE CHAIN REACTION

Total RNA was extracted from fresh feces as described previously (Nakamura et al., 2005). Primers and reverse transcriptase-polymerase chain reaction (RT-PCR) conditions were described elsewhere (Kitajima et al., 2009). PCR products were separated by 2% agarose gel electrophoresis.

### SEQUENCE ANALYSIS

Polymerase chain reaction products were sequenced as described previously (Ohsugi et al., 2004). Nucleotide sequences were aligned using ClustalW. A phylogenetic tree was generated from a bootstrap analysis of 1,000 replicates using the neighbor joining method. Evolutionary distances were computed by the p-distance method using MEGA 5.05 software (Tamura et al., 2011).

## RESULTS

### IDENTIFICATION OF MICROBIOLOGICAL STATUS IN MICE REARED UNDER SPF OR CONVENTIONAL CONDITIONS

**FIGURE 1:**
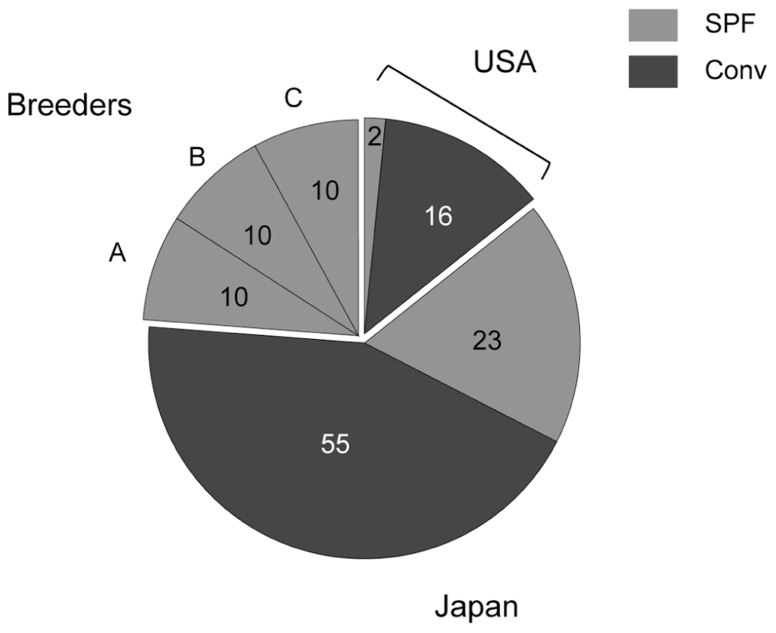
**Numbers of tested mice**. Two SPF and 16 conventional mice derived from US animal facilities, 23 SPF and 55 conventional mice derived from domestic facilities, and 10 of each SPF female C57BL/6 mouse obtained from breeders A, B, and C were tested in this study.

Microbiological analysis was performed in 96 mice from 29 animal facilities, including 18 mice from five US facilities. In addition, 10 of each SPF female C57BL/6 mouse were purchased from breeders A, B, and C (**Figure 1**). Most of the tested mice were negative for viruses and bacteria (**Table 1**). *Mycoplasma pulmonis* was isolated from a single mouse from a Japanese conventional facility. *Trichomonas* spp. contamination was common in US facilities, despite their disparate geographic locations across the country. Almost all of the mice reared under conventional conditions were positive for parasites and protozoa.

### ANTIBODIES TO MNV IN ANIMAL FACILITIES IN JAPAN AND THE US AND COMMERCIAL MICE IN JAPAN

**FIGURE 2:**
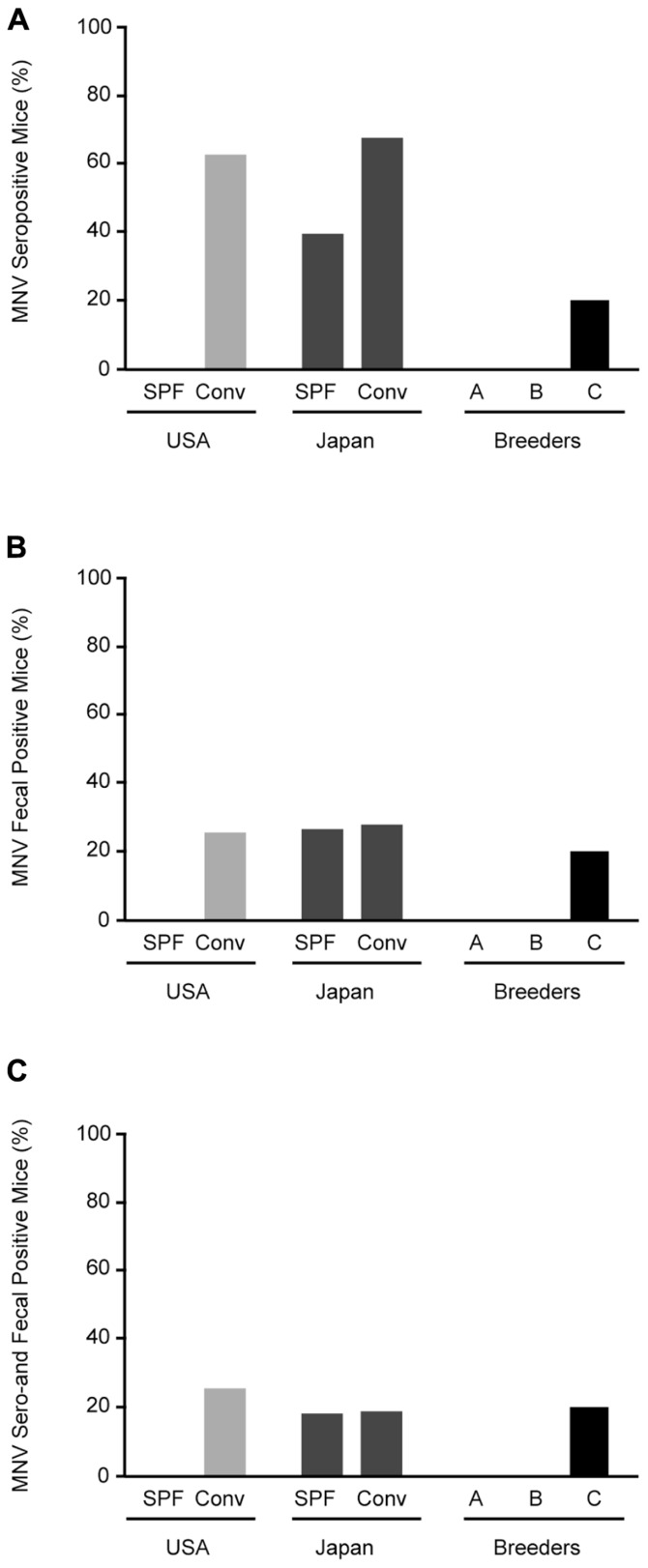
**Prevalence of MNV infection in conventional and SPF mice.** **(A)** MNV seropositive mice from animal facilities in Japan and the US and breeders in Japan. **(B)** RT-PCR detection of MNV sequences in feces from tested mice. **(C)** Mice positive for both MNV antibodies by ELISA and MNV-specific sequences in feces by RT-PCR. SPF, specific pathogen-free; Conv, conventional.

Specific pathogen-free mice from the US were negative for MNV, whereas 62.5% (10/16) of conventional mice from US animal facilities were positive for MNV (**Figure 2A**). MNV infection was found in 39.1% (9/23) of SPF and 67.3% (37/55) of conventional mice in Japan. Surprisingly, antibodies were found in 20% (2/10) of commercial SPF mice derived from breeder C.

### DETECTION OF MNV IN FECES FROM SPF OR CONVENTIONAL MICE

Stool specimens were collected from 126 animals from 32 separate laboratory colonies in Japan and the US and screened for MNV (**Figure 2B**). No MNV genome segments were isolated from SPF mice from the US, similar to the serological study, whereas MNV was isolated from 25% (4/16) of conventional mice. MNV was isolated from 26% (6/23) of SPF mice and 27.3% (15/55) of conventional mice from Japan. The frequency of MNV isolation was similar between SPF and conventional mice from Japan and the US. In commercial SPF mice, MNV was isolated from 20% (2/10) of the mice from breeder C. The mice positive by both serological and molecular biological methods are shown in **Figure 2C**. These results suggest that MNV infection is widespread in both conventional and SPF mice, including commercial mice in Japan.

### FREQUENCY OF DETECTION OF MNV

Five mice were autopsied 4 weeks after MNV-specific sequence detection and tested for antibodies against MNV (**Table 2**). All tested mice had antibodies for MNV. The MNV-specific sequence was not always detected in the feces of the mice except mice D and E during tested 4 weeks. The sensitivity of RT-PCR did not improve with different PCR conditions, PCR reagents, or primers. Thus, we speculated that the amount of MNV particles in the feces might vary in each mouse due to, for example, host defenses or intestinal circumstances.

**Table 2:** Change of MNV detection from feces after the first isolation.

Mice	Week after detection of MNV					ELISA
	0	1	2	3	4	
A	+*	+	-	-	-	+
B	+	-	-	+	-	+
C	+	-	-	-	-	+
D	+	+	+	+	+	+
E	+	+	+	+	+	+

* RT-PCR positive.

### PHYLOGENETIC ANALYSIS

**FIGURE 3:**
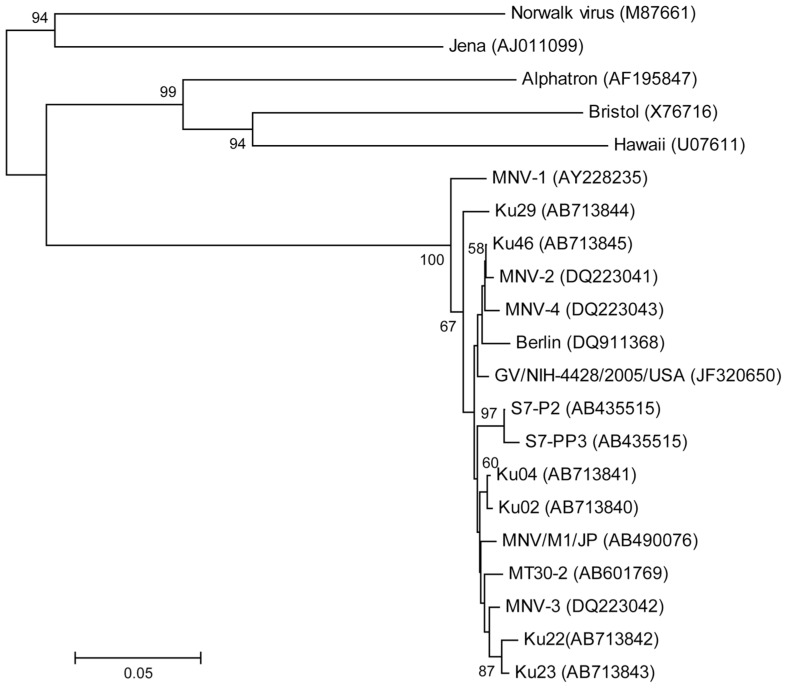
**Phylogenetic analysis of the partial capsid gene of norovirus (approximately 350 nucleotides).** Phylogenetic relationships were inferred using the neighbor joining method, with the evolutionary distances computed using the p-distance model. The statistical support for tree nodes was evaluated by bootstrap analysis (1,000 replicates). Bootstrap values (>50%) are indicated at branch nodes. Scale bar indicates nucleotide substitutions per site. Numbers in parentheses indicate GenBank accession numbers. Human isolates, Norwalk, Alphatron, Bristol, and Hawaii; bovine isolate, Jena; North American isolates, MNV-1, -2, -3, and -4, GV/NIH-4428/2005/USA, Ku22, and Ku 23; European isolate, Berlin; Japanese isolates, S7-P2, S7-PP3, MNV/M1/JP, MT30-2, Ku02, Ku04, Ku46, and Ku29 (SPF mice).

The partial nucleotide sequence was determined for the 5′ terminus of the capsid gene (**Figure 3**). Phylogenetic analysis of the partial capsid sequences of MNV showed widespread expression in various facilities throughout Japan and the US, and that these virus strains comprise reported MNV subgroups. Reference strains MNV-1 and Ku29 derived from an SPF facility in Japan were divergent from the other strain group including reference stains MNV-2, -3, and -4. Ku02 and Ku04 derived from the same facilities in Japan exhibited moderate similarity, but the sequences of Ku22 and Ku23 derived from different conventional US facilities were closely related despite their disparate geographic locations across the country.

## DISCUSSION

In this study, we analyzed MNV infection in mice derived from animal facilities in Japan, the US, and three breeders (Japan) using serological and molecular techniques. We show that MNV infection is widespread among both conventional and SPF mice in Japan, as well as commercial SPF mice. In a previous study, serological analysis revealed that 53.4% of conventional mice are positive for MNV, 48% positive for mouse hepatitis virus, and 31.2% positive for *M. pulmonis* (Kitagawa et al., 2010). Consistent with this report, we show that MNV is the most prevalent infection in mouse laboratory colonies, and the mice we tested, with the exception of one case, were negative for the antibodies for hepatitis virus and *M. pulmonis* (**Table 1**). This report is the first on MNV infection in SPF mice in Japan and commercial mice.

The frequency of MNV infection as detected by RT-PCR was lower than that detected by serological study using ELISA. We detected MNV antibodies in all five mice tested 4 weeks after the first MNV sequences were detected by RT-PCR Using RT-PCR, MNV sequences were detected consistently in the feces of only two mice. Thus, a survey for MNV infection might require the use of serological methods, such as ELISA. The first norovirus to infect mice, MNV-1, caused death in severely immunocompromised mice lacking recombination-activating gene 2 (RAG2) and signal transducer and activator of transcription 1 (STAT-1; RAG2/STAT1^-^^/^^-^) and mice lacking both the alpha/beta interferon (IFN-α/β) and the IFN-γ receptors (IFN-αβγR^-^^/^^-^; Karst et al., 2003; Mumphrey et al., 2007). In this study, the mice infected with MNV exhibited no clinical signs, though the tested mice, with the exception of the mice from breeders, were genetically modified, including compromised immune systems. Recently, MNVs (except MNV-1) were associated with asymptomatic infection and shedding in both normal and genetically modified mice (Hsu et al., 2006; Muller et al., 2007). The origin of MNV in laboratory mouse strains is unknown. Phylogenetic analysis of the partial capsid sequences of MNV in this study showed that the isolated virus strains comprised the reported MNV subgroups, though MNV-1 and Ku29 isolated from the SPF facility were divergent form the main group. However, bootstrap values for the position of Ku29 were not high (<70%). Thus, further study is needed to address whether the Ku29 isolate belongs to a new MNV genotype. These results indicate that attenuated (persistent) strains of MNV, such as MNV-2, -3, and -4, are spread around the world, making the viruses difficult to detect in animals without active screening (Muller et al., 2007; Barron et al., 2011). Using ELISA, we did not detect MNV antibodies in stocked serum from mice transported from domestic animal facilities before 2009 (data not shown). These results suggest that MNV has been spreading through 50% of animal facilities in Japan in only 2 years.

Finally, our results suggest that MNV infections have spread widely throughout the animal facilities in Japan, not only conventional mice colonies, but also SPF mice colonies. The findings warrant further studies to elucidate the spread of MNV infection in SPF colonies, including breeders, in Japan. These results also suggest that SPF laboratory mice, including commercial mice, which might require considerable attention as an animal model for human norovirus infection.

## Conflict of Interest Statement

The authors declare that the research was conducted in the absence of anycommercial or financial relationships that could be construed as a potential conflict of interest.
